# A Review of Lysimeter Experiments Carried Out on Municipal Landfill Waste

**DOI:** 10.3390/toxics9020026

**Published:** 2021-02-02

**Authors:** Dominika Dabrowska, Wojciech Rykala

**Affiliations:** Faculty of Natural Sciences, University of Silesia, Bedzinska 60, 41-200 Sosnowiec, Poland; wojciech.rykala@us.edu.pl

**Keywords:** landfills, leaching, lysimeters

## Abstract

The groundwater risk assessment in the vicinity of landfill sites requires, among others, representative monitoring and testing for pollutants leaching from the waste. Lysimeter studies can serve as an example of dynamic leaching tests. However, due to the bacteriological composition of the municipal waste, they are rarely carried out. These tests allow for the proper design of the landfill protection system against migration of pollutants into the ground, assessment of bacteriological, biochemical and chemical risk for the groundwater, determination of the water balance of leachate as well as examination of the course of processes taking place in the waste landfill with a diversified access to oxygen. This paper addresses the issue of performing lysimeter studies on a sample of municipal waste in various scientific centers. It analyzes the size of lysimeters, their construction, the method of water supply, the duration of the experiment, the scope of research, and the purpose of lysimeter studies.

## 1. Introduction

Along with the rapid development of industry and the world population, a large increase in the generation of municipal waste can be observed [1]. Due to the poor control and monitoring of that trend, a growing number of wild landfills are created worldwide, posing a problem for the natural environment. The environmental risk assessment is an analytical method for analyzing environmental impacts using historical data collection, identification of regional risk sources, and probability and impact estimation of signal risk type. The area of impact of the landfill on the environment is related to the type and number of substances collected in the landfill, their ability to move, bioavailability, the hazardous materials stored, the geological structure, or the type of protective devices used. Significant environmental hazards occur as a result of the impact of seepage water on the groundwater which may lead to groundwater pollution by hazardous substances [2].

Municipal waste tends to be highly diverse in terms of its morphological composition which depends on:

- seasons of the year–climatic variability;
- waste origin area–urban or rural;
- wealth of the local residents;
- ecological mentality of local residents.

Several methods of waste disposal are available, but the most frequently adopted procedure is to transport it to controlled landfills. However, the biodegradability of solid municipal waste varies depending on the designated place for its storage, season, climate, or cultural practice in the world [3]. A new approach to landfilling biodegradable waste was introduced in the strategy for reducing biodegradable waste going to landfills [4], which specifies that in 2010, no more than 75% of the total waste generated in 1995 will be deposited [5]. 

The collected waste undergoes three-stage biological and physicochemical processes, such as the mineralization of organic forms of nitrogen, sulfur, and phosphorus to inorganic forms, hydrolysis or fermentation, which give rise to the formation of new, environmentally hazardous chemical compounds [6]. The products of decomposition of the organic matter found in the waste lead to the formation of gases and leachate, which can migrate to aquifers due to the lack of adequate protection of landfills. Persistent organic pollutants (POPs), polycyclic aromatic hydrocarbons (PAHs), pesticides, pharmaceuticals, and cosmetics account for a large share of the risk for groundwater. Experimental studies of migration of such pollutants can be carried out using lysimeters.

In the traditional sense, a lysimeter is a device filled with soil for measuring the infiltration and evapotranspiration values in a natural hydrologic cycle [7]. Most often, the lysimeter is a column of materials resistant to moisture of a diameter in the range of 0.5–2.0 m and length in the range of 1–3 m [8], although it may have a different construction. There are three types of lysimeters: classic, weighing lysimeters, and tension lysimeters. The design of the second type is usually similar to the classic lysimeter, but it is expanded with weighing devices for more accurate studies of evaporation in dry periods [9]. This type of lysimeter facilitates agrotechnical research [10]. In turn, temperature-controlled tension lysimeters make it possible to control the temperature inside the lysimeter [11]. There are also two types of special lysimeters–the hillside lysimeter [12] and groundwater lysimeter. The former can be installed in the mountains and the latter allows for the determination of the dynamics of groundwater formation, in particular in alluvial soils. Further, microlysimeters merit special attention, as they are also used in the study of non-rainfall water input to measure evaporation [13].

Lysimeter studies form part of dynamic leaching tests [7] in which fluid (most often water) is added as a leaching medium at a given time and in a given volume. Given that lysimetric tests are not typically carried out on small soil samples and their duration significantly exceeds the time of traditional dynamic leaching tests, they are not the dominant type of tests. Lysimeter studies can be used in various fields of science, e.g., hydrogeology, agronomy, agrotechnics, ecology, environmental protection, geochemistry, and waste management [7]. They were initially conducted to investigate the sources and value of transpiration from plants [14].

The most important advantage of lysimeter studies lies in the fact that the experiments are conducted on undisturbed soil and are repeatable. In contrast, their main disadvantages include disturbed drainage, the costs of conducting reliable tests and limitations related to the size of tested soil samples [15].

It should be noted that the flow trajectories in unsaturated zones are mostly vertical. This means that the pollution processes may be tracked in advance where there are data available on the flow and transport in the aeration zone. The depth to the water table and the nature of the unsaturated zone are also important for the development of models, such as the soil moisture water balance model (SWAGMAN model), that estimate net recharge [16], since they describe the depth of the material through which a pollutant must migrate before reaching the aquifer. Moreover, the identification of contaminants in wells or piezometers becomes a late alert. Contaminants have a huge negative impact on groundwater and health safety. More attention should therefore be given to unsaturated zones, because the technology for groundwater reclamation does not offer a 100% chance of cleaning the aquifer to drinking water standards.

Lysimeter studies can also be useful in research connected with the spatial and temporal conditions of the shallow aquifer [17] providing data to the model. There are solutions, such as engineered barriers [18], which isolate contaminated soil and groundwater to keep them from mixing with clean groundwater. Such solutions can be the first step once a threat is adequately detected.

Lysimeter research may be mapped using flow and transport models for the unsaturated zone, such as HYDRUS software [19], which are helpful in the assessment of aquifer vulnerability.

Lysimeters can also serve as experimental tools for studying the environmental processes in the waste under controlled conditions, and the results of such studies can provide a good basis for a risk analysis. The first steps aimed at exploring the potential of landfill bioreactors to improve waste stabilization, leachate treatment, and landfill gas production were made as early as 1970. Several lysimeter and full-scale laboratories were then located in the United Kingdom, the United States, Germany, and Australia [20].

Moreover, the results of lysimeter studies may form the basis for statistical analyses involving artificial intelligence. Artificial neural networks [21,22,23] constitute a simulation method capable of imitating extremely complex functions or even modeling poorly defined physical processes. Those networks may be used in classification and regression studies. Given that this method is also suitable for monitoring phenomena occurring in real time [24,25,26], it appears to be ideal for the development of a model of the spreading of pollutions leaching from the waste.

## 2. Basic Assumptions Underlying Municipal Lysimeter Research

**Figure 1:**
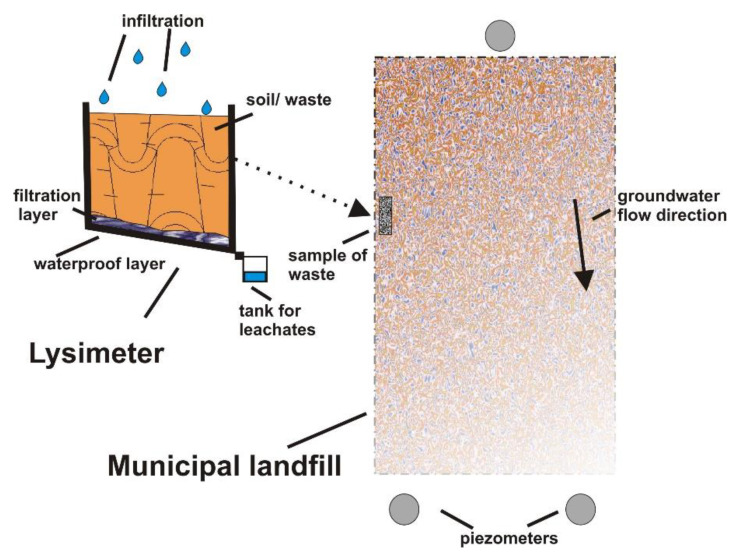
General sketch of the lysimeter.

The lysimeter studies on the border between hydrogeology and waste management are used to determine the impact of the waste on the environment. with account being taken of the kinetics of physicochemical processes occurring at the landfill site. A general diagram of the lysimeter used for lysimeter studies on municipal waste along with the location of a typical sampling site (their size) is shown in Figure 1. The purposes of that type of lysimeter research require that the size and intensity of eluted pollutant loads and their characteristics are also taken into consideration, allowing for the assessment of the degree of threat to the groundwater posed by the waste. 

Lysimeter tests on municipal waste are the least frequently performed experiments due to the fact of their duration, costs, and specificity of waste. In general, the test results are to determine the degree of pollution associated mainly with the operation of the landfill or the approximate possibility of its occurrence in a nearby natural environment [27].

In lysimeter studies conducted on municipal waste, the technical design of the device is less important than the purpose of the research. The simplest example of the use of lysimeter is to determine the quality of the leachate [28]. More complex and targeted lysimeter studies include, among others, research on the migration of specific pollutants and risk assessment, testing recirculation of leachates and its influence on waste, testing biogas accumulation [29], determination of different waste storage methods [30], and settlement and geotechnical characteristics of municipal solid waste [31]. Basic information on selected lysimeter tests is presented in Table 1. The intensity and application of lysimeter studies result from the aims and general outlines of projects and vary between different countries.

## 3. Application of Lysimeter to Determine the Quality of the Leachate

The results of one of the first lysimeter experiments were presented in the work of Pohland (1975) [32]. The study evaluated changes in the chemical composition of the leachate. The experiment lasted 699 days. Wastes of a density of 319 kg/m^3^ were placed in open and sealed cells where the moisture of the wastes was also observed [32,33]. Very similar studies were performed by Kemper and Smith (1981) and Reinhart (1995) [34,35].

A more recent example of lysimeter testing is an experiment at a landfill in Bangladesh. The purpose of that study was also to determine the leachate quality for the appropriate planning and design of landfills and sewage treatment plants. Three lysimeters were designed with an external diameter of 1.98 m and an internal diameter of 1.48 m, a height of 3.35 m, and a leachate tank (3.68 m × 1.56 m × 1.64 m) containing four separate pipes for draining leachate to containers for temporary collection and storage. The lysimeters were built using a 250 mm thick brick wall erected on a reinforced concrete foundation mat at a depth of 760 mm. At the bottom of each lysimeter, a 125 mm thick layer of concrete was placed, and then the lysimeters were filled with stone chips (diameter 5–20 mm) and coarse sand (diameter 0.05–0.4 mm) to a height of 15 cm each to ensure homogeneity [36]. In the first lysimeter, aerobic conditions and open dumping were simulated, whereas sanitary anaerobic conditions existed in the second lysimeter. The third lysimeter was analogous to the second, except that the 900 mm thick natural topsoil was used instead of the 300 mm a conventional compacted clay (CCL) and 600 mm thick topsoil.

The results of the Bangladesh study are presented for several pollution indicators. The greatest variation in pH was observed in the sanitary landfill lysimeters with a gas collection and leachate recirculation system with cap liner-II (900 mm thick natural topsoil). The highest values were recorded in lysimeter A—8.47. The alkalinity up to week 23 of the experiment ranged between 1111.2–9000 mg/L for lysimeters B and C. The diversity of alkalinity is related to the difference in the thickness of the cap liner and compaction conditions in the two lysimeters. The potassium concentration in the initial work of the lysimeter was 1650.5 and 185.8 mg/L, and after 50 days, it declined significantly to 855.9 and 1566.8 mg/L. After 500 days, the maximum values of 1370 and 2590 mg/L were reached for leachate detection (A1) and the collection system (A2), respectively. The calcium content ranged between 253.5–789.9 mg/L, 217.9–664.2 mg/L, 153.0–578.0 mg/L, and 140.0–664.4 mg/L for A1, A2, and lysimeters B and C, respectively. The sodium concentration was 1267.9–2424 mg/L, 1251.6–2369.6 mg/L, 1256.3–2764.3 mg/L, and 1011–24,249.1 mg/L for A1, A2, B and C, respectively. The copper concentration was 0.04 mg/L. The iron content in the lysimeter effluent reached a maximum value of 72.0 mg/L.

A study conducted in 2020 in Canada [37] aimed to determine the water balance. Seven lysimeters of two granular media types (i.e., topsoil and compost mixture) and three types of vegetation 15 (i.e., native grass species, alfalfa, and Japanese millet) were constructed. The lysimeters had dimensions of 1.8 m × 1.8 m × 1.1 m and were constructed by placing an 80-cm layer of biocover material over a 30-cm layer of washed uniform coarse gravel. The CH_4_ at a pressure of 34.5 kPa was supplied to the lysimeters to simulate landfill conditions. The bottom of the lysimeters was sloped. Micrometeorological data (i.e., precipitation, maximum and minimum air temperature, wind speed and direction, solar radiation, and relative humidity) as well as soil moisture and vegetation data were monitored at the research stand.

Lysimeters with a compost mixture and topsoil vegetated with Japanese millet transmitted lower percolation in comparison to lysimeters with native grass species and alfalfa. The lysimeter with alfalfa had the highest average evapotranspiration. The results of the modeling carried out in that experiment with the use of HYDRUS-1D software require further analysis.

## 4. Application of Lysimeter to Simulate Different Landfill Conditions and Gas Production

In India, lysimeters were also located in landfills. They served as a basis for the determination of the benefits of operating a landfill with and without recirculation of leachate in tropical weather conditions. New and old landfills were simulated to show the difference in the fresh and old municipal waste. Four lysimeters were built using reinforced concrete rings. The lysimeters were constructed as follows:

- R1—it was filled with fresh municipal solid waste to simulate a young controlled landfill without leachate recirculation;
- R2—it was filled with fresh municipal solid waste to simulate a young landfill, a bioreactor with leachate recirculation;
- R3—it was filled with mined municipal solid waste to simulate an old controlled landfill without leachate recirculation;
- R4—it was filled with mined municipal solid waste to simulate an old landfill bioreactor with leachate recirculation [38].

The R1 and R2 lysimeters were put into service two months prior to the others. Moreover, coarse gravel was placed at the bottom of the lysimeter to a height of 0.2 m as a drainage layer. The substrates were manually compacted using a compactor. Suitable polyvinyl chloride (PVC) pipes were fitted below the gravel drainage to facilitate leachate collection. The PVC gas collection pipes were also installed to facilitate the monitoring of methane in biogas emissions.

In the experiment performed in India, the solid municipal waste contained less moisture (27% to 28%) and volatile substances (30%) than the fresh waste. It is a very low moisture content detrimental to any microbial activity. The obtained results of the study on “settlement patterns” show that higher humidity and lower density mean that fresh waste settles faster than extracted waste. The volume reduction calculations resulting from the distribution of organic solids were 13.7%, 18.6%, 7%, and 10.3% in R1, R2, R3, and R4. Besides, the role of climate in determining the settlement of waste should not be overlooked. The average effluent efficiency was 8 L/m^2^ during the first 4 months of operation of the R2 lysimeter. After that period, the amount of leachate produced increased due to the rainfall. The collected effluent was recycled again, and the average efficiency increased to 11 L/m^2^. Out of the large amount of leachate collected in the lysimeter, one liter of the leachate was used as a weekly sample, while the remaining volume was placed back in the lysimeter. During the recirculation, due to the lower absorption capacity, higher cumulative amounts of leachate were observed in the old lysimeter storage site rather than in the new one. It should be emphasized that the landfill field capacity decreased with the degradation of organic fractions which contribute to the majority of waste capacity absorption and increasing density resulting from the disappearance of pores available for migration and moisture retention. The leachates from the old lysimeter storage sites showed a higher conductivity up to 53 S/cm. It was similar to the concentrations of two main inorganic ions—sodium and chlorides. Their values were initially higher in the leachates from R3 and R4. High values of dissolved solids were initially observed in the leachates from R1 and R2. In the bioreactor (R2), a decline in dissolved solids content from the initial level was observed after four months of operation. As regards the total and volatile solids, the reduction was 57% and 75%, respectively, which was observed in R2, while the solids content remained unchanged in R1. The content of methane produced in the R2 and R4 lysimeters was 34–59% and 15–47%, respectively, whereas in the controlled landfills R1 and R3, there values were 16–35% and 10–16%. The lower methane concentrations in the controlled discharges are seen as proof of leaching, indicating the inability of the system to develop active methanogen. It can also be stated that the production of methane gas increased and concentrated during the active life of the landfill. Furthermore, the in situ recirculation was sufficient for leachate management when using lysimeters in tropical weather conditions. The inorganic accumulation of components as a result of effluent recirculation requires the application of additional amm-N and chloride removal methods.

An experiment carried out in China examined the performance of simulated waste components at different biogas accumulations. One of the main problems in the production of biogas is the formation of leachate in landfills, which can result from low permeability, high humidity, or great height of the landfill. Furthermore, high leachate levels can cause landfill instability, e.g., damage to the slopes. The biogas is collected at the landfill by the discharge system in the upper direction, while the leachate flows downward under gravity into the discharge system. Two stainless-steel lysimeters were built for testing. Each lysimeter consisted of a stainless-steel cylinder 75 cm high, 18 cm in internal diameter, a compression plate (perforated plate of stainless steel with an identifier smaller than 18 cm), a fixed cap (acrylic plate), a compression unit equipped with a hydraulic cylinder, and a hand pump with a pressure gauge [39].

In the Chinese experiment, the characteristics of biogas production changed during the experiment, depending on the concentration of the waste and the method of collecting the biogas. Both the lysimeter collecting biogas from the top port only (LT) and the lysimeter collecting biogas from both the bottom and top ports (LTB) showed a similar pattern of methane production before compaction. Methane production in the LT and LTB lysimeters reached a maximum level: 2.6 L/day on day 32 and 2.9 L/day on day 30, respectively. The increase in biogas production under compressed conditions was explained by an increase in the surface area of contact between the biomass and the improvement of the conditions of substrate transfer. At the end of the experiment, the daily methane production from LT dropped below 0.2 L/day. The compaction in LTB immediately reduced the methane production rate from the upper port to 0.8 L/day but increased the methane production rate from below. Different gas collection practices affected the leachate generation in the simulated landfills. In LT and LTB, the volume of the pores declined as a result of compaction. The total amount of effluent removed from LT after concentration was similar to LTB. The compaction in LT and LTB resulted in the production of 877 mL and 1506 mL of leachate, respectively. The leachate both in LT and LTB showed high concentrations of chemical oxygen demand (COD) and VFA (Volatile fatty acids) during aeration and at the beginning of the anaerobic phase. For compaction, COD and VFA levels changed in both cases. During the high daily production of methane, the COD and VFA levels fluctuated between 45 g/L and 60 g/L and between 25 g/L and 39 g/L in LT. The average water content in both lysimeters was 35%.

The study conducted in Jordan aimed to monitor the potential for solid waste emissions using an anaerobic lysimeter. A 100 L stainless-steel lysimeter was built as a reactor. The internal diameter and height of the reactor were 0.40 and 1.00 m, respectively. A perforated plate was attached at the bottom to prevent clogging, loss of the waste, and drainage of the produced effluent. The lysimeter made it possible to assess leaching, recirculation of leachate, precipitation simulation, detection of leachate, and the measurement of biogas content and volume [3]. The results obtained from the lysimeters were intended to improve the design and operational parameters of the waste storage methods. Besides, the experiment addressed the issues of developing recommendations on the sustainable operation of landfills, current practices in the field of waste storage in the country, and the effects of climate change.

At the beginning of the experiment in Jordan, methanogen production showed an upward trend. However, after 260 days of testing, the gas and leachate extraction became negligible. After 160 days of detecting the first CH_4_, 45% of the waste showed a slow increase in methanogens. It was found that the methane content of the lysimeter was greater than 45%, which could lead to the risk of explosion and self-ignition at the landfill. The CO_2_ from waste decomposition had a biogenic origin and did not affect the total greenhouse gas emissions. The moisture content at the top of the lysimeter was 52% in the fresh waste and 43% in the digestate. The volatile solids content was 45% in the fresh waste and 32% in the digestate. During the rainy season, an increase in the production of leachate from the waste was observed and amounted to 10.2, 14, and 15.7 L after 125, 175, and 200 days. In contrast, in the dry season, only a small amount of leachate was produced, approximately 2.5 L. The COD concentration initially increased to 40,000 mg/L in the first stage, then carbon compounds in the leachate were used for methane production and the amount of COD decreased to 982 mg/L. The leachate recirculation during the phase stabilization on days 0–50 contributed to an improvement in efficiency in relation to biological activity and methanogenesis. The concentration of metals in the effluent was high, that is 0.2, 1.08, and 0.4 mg/L for Cu, Ni and Cr, respectively, during the stabilization and dry period up to 111 days, yet it was lower in the rainy season up to 202 days, when it declined to 0.1, 0.4, and 0.06 mg/L.

Note should also be taken of a simulation experiment carried out in Italy aimed at storing residual stabilized waste from a landfill to test its emissivity. Two samples were taken from a mechanical biological treatment plant located in northern Italy. The former was analyzed immediately after collection, the latter was aerated for a long time in a lysimeter reactor. The lysimeter was a 300 mm diameter column with a total volume of 2 m^3^, built for simulation enabling natural air circulation [40].

The dominant fraction in the lysimeters in Italy was the under-sieve fraction (44% in the fresh sample and to 77% in the stabilized sample). More than 55% of carbon, 53% of nitrogen, 33% of sulfur, and 90% of heavy metals which were initially present in the fresh waste samples were landfilled. Given their highest residual emission potential, the most persistent elements and compounds were chlorides, sulfates, nitrates, and humic substances. The lysimeter experiment showed that the solid waste is not a good sink for chlorine, as 87% of it was progressively leached away, leaving 2.9 g/kg TS of Cl in the stabilized sample.

In Poland, lysimeter studies were performed by two research centers—the University of Silesia [41] and the Lodz University of Technology [42]. In the first case, the tests were carried out in two 230 L lysimeters filled with waste. The aim of the study was to determine the total volume of the obtained effluents, the values of specific electrolytic conductivity, temperature, pH, Eh, and the characteristic indicators of groundwater pollution in the area of municipal waste landfills. As part of the tests, microbiological and biochemical tests were also carried out. The lysimeters were constructed of PVC with a diameter of 0.42 m and a height of 1.8 m. The second experiment used three lysimeters of the working volume of 15 L. The aim of the study was to evaluate changes in the waste, leachate, and gas as well as to compare the processes under aerobic and anaerobic conditions. The lysimeters consisted of a PVC cylinder of 0.15 m in diameter and 1.15 m in height.

The results of the pilot phase of the lysimeter experiment conducted in Poland at the University of Silesia show that the electrolytic conductivity value in leachates is approximately 30 mS/cm. A gradual elution in concentrations of most components was observed. The microbiological analyses performed for those leachates showed that the number of heterotrophic bacteria varied significantly. The leachates were contaminated with, among others, coliforms and enterococci from groups including *Salmonella* sp., *Shigella* sp., *Pseudomonas* sp., *Proteus* sp., and *C. perfringens*. The highest number of enterococci reached the level of 53,000 cells/100 mL. The microbial population in the leachates from the winter period had a higher metabolic activity and was more diverse in comparison to those from the spring, which was explained by the substrate quality and availability of nutrients in the waste during winter and spring.

The simulations of aerobic landfills in the experiment performed in Poland at the Lodz University of Technology indicate that recirculation of leachate influenced the composition of leachate indices and the formed gas. A higher recirculation rate caused a 75% reduction in COD after 17 days, whereas at a low recirculation rate, the same reduction of COD in the leachate was achieved after about 100 days. The O_2_ assimilation and faster CO_2_ production in the aerobic lysimeters were observed in the lysimeters with a low recirculation rate. Better waste stabilization occurred in aerobic lysimeters, which was confirmed by the amounts of carbon used during the aerobic and anaerobic processes (it was reduced by 26.5% in the lysimeter with a high recirculation rate and by 31.8% in the lysimeter with a low recirculation rate).

Another example is a lysimeter study carried out in Turkey [43]. The construction of the test cells based on an earlier lysimeter experiment described in the work of Bilgili et al., 2006 [44]. Four identical field-scale test cells simulating anaerobic (AN-1), anaerobic leachate recirculated (AN-2), semi-aerobic (A-1), and aerobic (A-2) landfills were taken into account. These test cells were constructed with the dimensions of 20 × 40 × 5 m. Each of the cells had the impermeable liner and the leachate collection pipe. In the semi-aerobic test cell, this pipe was also used to provide aeration. The purpose of the study was to assess the impact of aeration, semi-aeration, and leachate recirculation on the biological degradation rate, taking into account the quantity and quality of leachates.

In the lysimeter tests carried out in Turkey [43], the amount of the produced effluent was: A-1 154 m^3^, A-2 186 m^3^, AN-1 111 m^3^, and AN-2 141 m^3^. At the same time, the amount of the reduced effluent was: A-1 117 m^3^, A-2 76 m^3^, and AN-2 55 m^3^. The rapid reduction in the effluent in test A-2 was due to the evaporation and the right temperature.

In the course of the experiment, pH measurements were performed. Approximately, throughout the duration of the test, the pH was below 7. This was due to the organic acids that were formed during the biodegradation of organic matter in the solid waste. The time needed to stabilize the pH in the AN-1 and AN-2 tests was 100 and 50 days, respectively.

The production of methane during the test occurred with a simultaneous decrease in the sulfide content. Initially, the sulfide concentrations were approximately 1500 mg/L in tests, then, after about 200 days, they fell below 200 mg/L. Under anaerobic test conditions, the initial sulfide concentration was 1500 mg/L. After 100 days of transition into the methanogenic phase, the sulfide content began to decrease rapidly.

The experiment described in detail the changes in the value of ammonia, nitrates and COD (as the ratio of total inert COD non-biodegradable soluble + non-biodegradable particulate). Initial ratios were at very low levels in all test cells and began to increase after 20 days of operation. The COD ratio reached 55% on day 200 in A-1 and A-2 and 50% after 375 and 200 days of operation in AN-1 and AN-2 [43]. Ammonia is one of the main pollutants in the leachate from landfills, in particular the new ones, due to the deamination of amino acids during the destruction of organic compounds. Ammonia values decreased from 1850 to 125 mg/L during the experiment, and nitrate concentrations were in the range of 100–200 mg/L and 50–100 mg/L for A-1 and A-2, respectively.

The experiments conducted in Reference [45] aimed to define the stabilization performance of a semi-aerobic landfill under conditions of different water availability and waste content, as well as the performance of a semi-aerobic landfill under tropical wet and dry climate conditions. Six lab-scale lysimeters with two different types of waste (with low putrescible and high putrescible content) were used. The lysimeters were 1.0 m in height, their inner diameter was 40 cm. Among the most important parameters, pH, alkalinity, TS and VS, volatile fatty acids, COD, TC and TOC, BOD5, nitrogen compounds, and chlorides in the lysimeters were measured. 

A better efficiency of the landfill was achieved in dry climatic conditions. The landfill stabilization day followed the application of a new layer of waste in each climatic season. The experiment proved that once stabilized, the lower layer of the landfill acts as an internal biological filter. The best performance for the semi-aerobic process was achieved at a water availability of approximately 1.5–2.4 kgH_2_O/kgTS, using waste with high putrescible content and no addition of external water.

## 5. Application of Lysimeter to Determine the Migration of Specific Pollutants and Risk Assessment

An experiment of that type was reported in China [46]. The aim of the study was to determine the migration and risk of the leaching of antimony content in three representative places. The lysimeter experiment used three characteristic soils–isohumosol, ferrosol, and primosol. For the purposes of scientific work, three lysimeters were installed at the Chinese Research Academy of Environmental Sciences. The containers were the core of the soil leaching equipment; however, the structure of the soil profile was simulated by filling the containers with different layers of soil. The container had the following dimensions: length 1 m, width 1 m, and height 1.6 m. The lysimeters were made of 12 mm thick carbon steel with 3 mm isobutylene rubber, a lining, and four square holes. The side had an 18 cm wall container for measuring the potential for oxidation reduction and a temperature sensor. Porous ceramic cups for collecting the soil solution were attached 5, 10, 20, 30, 50, 80, and 110 cm below the soil surface [46].

In all the soils in the lysimeter in China, Sb concentrations were very high in the surface layers (0–6 cm), but a sharp drop was observed below. The Sb content in the surface layers constituted 78–95% of the total content in the lysimeters. It should be stressed that Sb migrates very poorly in the soil. Sb was not detected in the leachate in primosol and isohumosol; however, it was found in the ferrosol solution and its concentration was 0.45 μg/L after leaching for 90 days, whereas after 120 days it was no longer detected. The Sb concentration in the effluent was very low. Detectable Sb in the ferrosol effluent was correlated with more leaching (1400 mm). This resulted from heavy rainfall on the soil with a high level of moisture, which facilitated the migration of Sb. The results of the study lead to the conclusion that Sb had low mobility in the three soils and higher pH values. Further, the salt content and rainfall could facilitate soil migration. The Sb was present mainly in the low solubility fractions that were associated with insoluble Fe and Al oxides. It appears necessary to continue the research in order to predict Sb migration in the future.

An experiment carried out in Korea [47] was aimed at simulating an environment similar to the Sudokwon landfill site. The changes in moisture, ash content, and combustibles values in the lysimeter were examined as well as an elemental analysis for C, H, O, N, and S was performed. Chemical analyses of leachates were also carried out. The experiment investigated the H_2_S production on seven different types of waste. The lysimeters had a volume of 100 L, sensors for measuring parameters were installed, a leachate spill tube and pebbles were used as a filter layer.

The experiment proved that the C&D debris waste, domestic waste, and industrial waste being landfilled cause the highest production of odors. The moisture, carbon, and sulfur contents in brick construction wastes were 0.01%, 3.24%, and 0%, respectively. COD_Cr_ and SO_4_ concentrations in the leachate were 660–4951 and 7–680 mg/L, respectively.

Another experiment carried out in Italy [48] adopted a phytotechnological approach through the use of living plants for the treatment of landfill leachate. In the study, poplar and willow grown in mesocosm were tested for their ability to remove specific pollutants from different amounts of leachates. 26 lysimeters were set up in the region of a landfill. Lysimeters consisted of a 1 m^3^ polyethylene box set upon a 0.3 m tall wooden bench with a 0.20 m high layer of gravel. EC, pH, BOD5, COD, chlorides, sulphite, fluoride, and total P were tested for leachate surfactants, NH_4_–N, NO_2_–N, NO_3_–N, and total phenols. 

The most important result of the study was that both species were able to treat up to 4950 m^3^ ha^−1^ in the second year of the project. Both species yielded the same aboveground biomass, but under high leachates treatment, poplar had better results. The content of parameters, such as BOD5, COD, and As, decreased, but in the case of Cl, surfactants, and NO_3_–N, no decrease in concentrations were observed.

## 6. Conclusions

Lysimeters have been used in hydrology, hydrogeology, agronomy, agrotechnics, and other environmental studies. In this study, 52 publications dealing with lysimeter research, in particular those concerning behavior and processes of numerous landfills from 1975 to 2020, were subject to review. A review of those publications showed that the lysimeter research can be successfully used to assess the physical, chemical, and biological characteristics of leachates, assess the leachate recirculation combined with precipitation and calibrate and test numerical models of water flow and chemical transport. The lysimeter experiments can be used when multidisciplinary research and a simulated process of experimental municipal landfills, waste, leachate, and gas are needed. The analyzed papers describing lysimeter experiments on municipal landfill waste provide valuable guidelines for researchers on how to perform similar leaching tests on waste. A study carried out in China by Ma and colleagues [49] summarized and analyzed the available studies on MSW aerobic degradation. The article summarizes 41 different tests for municipal waste from 2003–2019.

Studies on waste itself are performed on small samples using static (batch) leaching tests because they are cheaper. Lysimeter studies, due to the fact of their duration and the size of the sample, make it possible to obtain more reliable results and carry out a more precise assessment of groundwater vulnerability. Such experiments allow for the reconstruction of the conditions existing in the landfills. Giving consideration to the size of lysimetric waste samples, the water budget and the temporal changes in the physicochemical parameters of the leachates from a lysimeter permits making a forecast on the impact of real landfills on groundwater.

It should be noted that lysimeter studies for municipal waste can be a perfect complement to the methods of predictive models [50], geophysical tests [51], an isotopic multi-tracer approach [52], or even to analyses of data from groundwater monitoring to assess the vulnerability of groundwater to pollution in the area of pollution sources. The significance of lysimeter studies is evident especially in relation to the existing methods.

The following suggestions can also be highlighted:

1. The start of lysimeter tests on a waste sample should be preceded by testing the morphological properties of the waste, as well as the properties of construction (e.g., liner system). The average sift composition of the waste can be determined. The moisture content, plastic limit, liquid limit, plasticity index, and shrinkage limit can also be assessed in the case of the construction elements.
2. The scope of research should take into account the potential pollution that can migrate from the waste as well as the climatic conditions in which the experiment takes place.
3. It should be noted that not only the size of the waste sample and its duration are important in the final assessment of the leachate quality but also the type of redox conditions prevailing inside the lysimeter and the manner in which the tests are carried out.

Although numerous highly interesting and relevant issues are still to be addressed, the presented examples of lysimeter experiments constitute a perfect base for future research involving, for instance, artificial intelligence.

## Figures and Tables

**Table 1:** Selected lysimeter studies in the context of purpose, duration, and specifications.

No	Lysimeter Specification	Duration	Aim of the Study	Reference
1	The container with a length of 1 m, width of 1 m, and height of 1.6 m	5 months	Analyzes antimony (Sb) distribution, solubility, and mobility into natural soils of China	Hou, H.; Yao, N.; Li, J.; Wei, Y.; Zhao, L.; Zhang, J.; Li, F. Migration and leaching risk of extraneous antimony in three representative soils of China: Lysimeter and batch experiments. *Chemosphere* 2013, 93, 1980–1988.
2	Three cylindrical lysimeter having an outer diameter of 1.98 m and inner diameter (ID of 1.48 m, with a height of 3.35 m	6 weeks	Analyzes the characteristics of leachate in pilot scale landfill lysimeter for inorganic and organic compounds as well as metal and heavy metal concentrations against their operational conditions based on statistical tool through statistical package for social science (SPSS) software	Ahsan, K.; Shaikh, M.; Rafizul, I.; Alamgir, M. Statistical Analysis of Leachate Characteristics in Pilot Scale Landfill Lysimeter. *International Journal of Advanced Structures and Geotechnical Engineering* 2014, 3, 283–292.
3	Three lysimeters with a volume of 15 l	239 days	The investigation of the effect of waste aeration on the dynamics of the aerobic degradation processes in lysimeters	Slezak, R.; Krzystek, L.; Ledakowicz, S. Degradation of municipal solid waste in simulated landfill bioreactors under aerobic conditions. *Waste Management* 2015, 43, 293–299.
4	Height 75 cm, inner diameter 18 cm, a compression plate (perforated stainless-steel plate with ID less than 18 cm)	223 days	The performance of simulated landfills with different biogas collection practices, including upward biogas collection only and both upward and downward biogas collection	Xu, Q.; Qin, J.; Ko, J. Municipal solid waste landfill performance with different biogas collection practices: Biogas and leachate generations. *J Clean. Prod.* 2019, 222, 446–454.
5	Landfill test cells with the dimensions of 20 m × 40 m × 5 m	450 days	Leachate recirculation and the impact of aeration on the waste decomposition rate by means of leachate quality and quantity in field-scale landfill test cells	Top, S.; Akkaya, S.; Demir, A.; Yildiz, S.; Balahorli, V.; Bilgili, M. Investigation of Leachate Characteristics in Field-Scale Landfill Test Cells. *Int. J. Environ. Resources* 2019, 13, 829–842.
6	1 m height, inner diameter of 40 cm	100 days	To investigate the effect of inverse conditions of landfilling	Grossule, V.; Lavagnolo, M.C. Lab tests on semi-aerobic landfilling of Municipal Solid Waste under varying conditions of water availability and putrescible waste content. *Journal of Environmental Management* 256, 2020.
7	1.0 m height, inner diameter of 40 cm	6 months, divided into two subsequent phases	To investigate the performance of semi-aerobic landfill under tropical dry–wet climate conditions and to assess the potential benefits afforded by appropriate management of water input when operating the landfill by overlaying a new layer of waste in each climate season	Grossule, V.; Lavagnolo, M.C. Optimised management of semi-aerobic landfilling under tropical wet-dry conditions; Multidisciplinary. *Journal for Waste Resources & Residues*. 2020.
8	1.8 m × 1.8 m × 1.1 m	1 year	To evaluate the water balance performance of Evapotranspirative Landfill Biocovers (ET-LBCs) under Canadian cold-climate conditions	Jalilzadeh, H.; Hettiaratchi, J. Effect of Soil Type and Vegetation on the Performance of Evapotranspirative Landfill Biocovers: Field Investigations and Water Balance Modeling. *Journal of Hazardous, Toxic, and Radioactive*. 2020.
9	1 m^3^ polyethylene box set upon a 0.3 m tall wooden bench	2 years	To investigate the ability of poplar and willow grown in mesocosm to withstand and remove specific pollutants	Nissim, W.G.; Oalm, E.; Pandolfi, C.; Mancuso, S.; Azzarello, E. Willow and poplar for the phyto-treatment of landfill leachate in Mediterranean climate. *Journal of Environmental Management*. 2021.
